# Contributing Factors to Infectious Disease Risk and Vaccine Strategy Optimization in Cardiac Surgery Patients

**DOI:** 10.3390/vaccines14080686

**Published:** 2026-08-10

**Authors:** Monika Tokarczyk-Kloc, Julia Ciecierska, Robert Marguła, Katarzyna Herbetko, Bohdan Shmorhun, Leszek Szenborn, Mateusz Sokolski, Kamila Maria Ludwikowska

**Affiliations:** 1 Department of Pediatric Infectious Diseases, Wroclaw Medical University, Ludwika Pasteura 1, 50-367 Wroclaw, Poland; monikatokarczyk53@gmail.com (M.T.-K.);; 2 Faculty of Medicine, Wroclaw Medical University, Ludwika Pasteura 1, 50-367 Wroclaw, Poland; 3 Student Science Club of Transplantology and Advanced Therapies of Heart Failure, Faculty of Medicine, Wroclaw Medical University, Ludwika Pasteura 1, 50-367 Wroclaw, Poland; 4 4th Military Clinical Hospital, Rudolfa Weigla 5, 50-981 Wroclaw, Poland; 5 Clinical Department of Heart Transplantation and Mechanical Circulatory Support, Department of Cardiac Surgery and Heart Transplantation, Institute of Heart Diseases, Faculty of Medicine, Wroclaw Medical University, Borowska 213, 50-558 Wroclaw, Poland

**Keywords:** vaccine, cardiac surgery, immunization, vaccine-preventable infections, vaccine strategy, cardiopulmonary bypass, heart transplantation, infectious diseases

## Abstract

Patients undergoing cardiac surgery are particularly vulnerable to infectious diseases, which may adversely affect perioperative outcomes and long-term prognosis both before and after the procedure. Moreover, those requiring heart transplantation must take immunosuppressive medications, further compromising their immunity. This narrative review aims to look for the sources of increased risk for infections as well as synthesize vaccination recommendations for these patient groups based on the available literature and guidelines. We considered the influence of age, comorbidities, length of hospitalization, procedure-related risks, and blood product transfusions on the increased risk of vaccine-preventable diseases. By comprehensively addressing these factors, healthcare providers can develop tailored vaccination strategies that maximize protection for cardiac surgical patients while minimizing potential complications and optimizing overall health outcomes.

## 1. Introduction

Recent years have seen growing awareness of the role of vaccination not only in the prevention of infectious disease, but also as a means of reducing the risk of cardiovascular events and complications in cardiology and cardiac surgery patients.

Patients with chronic cardiovascular disease are considered a high-risk group for various infectious diseases, including coronavirus disease 2019 (COVID-19), influenza, respiratory syncytial virus (RSV) and invasive bacterial disease [1]. Among them, patients undergoing cardiac surgery constitute a particularly vulnerable subgroup, exposed to additional risks, such as cardiopulmonary bypass, blood transfusions and prolonged hospitalisation.

Despite the growing adoption development of less invasive endovascular procedures, the population of patients undergoing cardiac surgery addressed in this review has remained stable or even expanded in recent years. Due to the rapid development of modern surgical techniques, an increasing range of cardiac diseases can be treated surgically. According to the Adult Cardiac Surgery Database (ACSD), which gathers data from the majority of public hospitals in the United States, a total of 211,533 cardiac surgeries were performed in 2021. That data encompasses eight major procedures categories for which The Society of Thoracic Surgeons (STS) has developed risk-adjustment models: isolated CABG (coronary artery bypass grafting), isolated AVR (aortic valve replacement), isolated MVR (mitral valve replacement), MV (mitral valve) repair, AF (atrial fibrillation) correction surgery, aortic aneurysm and tricuspid procedure [2]. According to this database, the number of cardiac surgeries performed in the US remained relatively stable between 2010 and 2021, with a temporary decline observed in 2020 caused by the COVID-19 pandemic [2]. The most common among all performed procedures was isolated coronary artery bypass graft surgery (CABG) representing 72.4% of the total number of procedures in 2021. Among valvular heart defects, isolated aortic valve replacement was the most frequently performed surgery (16,873 cases, accounting for 7.9% of the total) [2]. Similarly, the National Adult Cardiac Surgery Audit (NACSA) reported a total of 25,764 cardiac surgery procedures performed across England, Wales, and Northern Ireland during 2022–2023 reporting period [3].

Another significant group requiring cardiac surgical procedures consists of patients with congenital heart defects. The National Congenital Heart Disease Audit (NCHDA) reported 11,407 procedures for patients with congenital heart disease (CHD) submissions among pediatric and adult patients in 2022/23. The most common congenital cardiac anomalies requiring surgery were tetralogy of Fallot; hypoplastic left heart syndrome (HLHS); transposition of the great arteries with an intact intraventricular septum (TGA-IVS) and atrio-ventricular septal defect (AVSD) [4].

These numbers presented above, together with the continued development of new surgical techniques that make cardiac surgery available to an increasing number of patients, mean that the population concerned is steadily expanding. Vaccines of particular relevance to this group include those against influenza, COVID-19, pneumococcal disease, tetanus, pertussis, RSV, and hepatitis A and B; in immunosuppressed patients after heart transplantation, vaccination against human papillomavirus (HPV) and herpes zoster (varicella-zoster virus, VZV) should additionally be considered.

In recent years, scientific societies issued consensus documents addressing both a specific patient group—heart transplant recipients—and the broader context of respiratory tract infection (RTI) prophylaxis in patients with cardiovascular disease, while a small number of countries, among them Canada, Australia, Taiwan and Poland, address the immunisation needs of this at-risk population in varying degrees of detail. Each of these documents covers only part of the problem, some being confined, for example, to respiratory infections alone, and they are inconsistent with one another: certain vaccines are included in some and omitted from others. This heterogeneity leaves the clinicians involved in the care of these patients—cardiac surgeons, cardiologists, infectious disease specialists and general practitioners—without a single point of reference, and in the absence of a common standard, immunoprophylaxis in this group remains largely dependent on individual judgement. As both immunoprophylaxis strategies and the molecular diagnosis of infection continue to evolve rapidly, there is a clear need for harmonised, evidence-based consensus recommendations on pre- and postoperative vaccine prophylaxis, covering the full range of vaccines relevant to this population.

This literature review summarises the current state of knowledge and the strategies proposed to date. It complements the existing literature by systematising the factors that may contribute to an increased risk of infectious disease in patients undergoing cardiac surgery and by setting out the specific circumstances that arise in the delivery of vaccination in this population, both in terms of need and of the possible precautions and contraindications, with the aim of providing a basis for a unified, consensus-based approach to immunoprophylaxis in this group.

## 2. Materials and Methods

This narrative review was based on a search of two bibliographic databases, PubMed/MEDLINE and Embase, initially conducted between April and November 2024 and updated in July 2026 to capture more recent publications and recommendations. Search terms, applied as both database index terms and free-text keywords, addressed the study population and vaccination, and included: “cardiac surgery”, “heart surgery”, “cardiothoracic surgery”, “cardiosurgical”, “coronary artery bypass”, “CABG”, “valve surgery”, “valve replacement”, “valve repair”, “vaccination”, “vaccine”, “immunisation”, and “immunization”.

The search was limited to publications from 2000 onwards to reflect contemporary vaccine formulations, immunisation schedules, and clinical recommendations. Sources comprised original research articles, case reports, and clinical practice guidelines concerning infectious disease risk and/or vaccination in adult patients undergoing cardiac surgery, available in full text in English or Polish. Records in other languages, without an available full text, with incomplete data, or published only as conference abstracts were excluded.

Retrieved records were exported to Mendeley Reference Manager and de-duplicated; titles and abstracts were screened first, followed by full-text assessment against the eligibility criteria, with screening organised thematically by author and a subsequent consolidated cross-check to ensure completeness, and any disagreements resolved by discussion.

Clinical practice guidelines were identified separately from the official websites of recognised scientific societies and public health institutions [ESC, EACTS, ACC, CDC/ACIP, WHO], with the most recent version issued by a recognised body selected in each case. Data were synthesised narratively; no meta-analysis or formal risk-of-bias assessment was performed, which is acknowledged as a limitation.

The primary list of chosen publications and online sources was proposed by K.M.L., M.T.-K., M.S., and approved by all the authors.

## 3. Factors Affecting Vaccination Strategies in Cardiac Surgical Patients

### 3.1. Age-Related Considerations

Cardiac surgical procedures most often involve two distinct patient groups: those within the first year of life with hemodynamically significant congenital heart defects, and those over the age of 60 undergoing surgeries for acquired cardiovascular diseases [2,4]. These age extremes represent well-established risk factor for infectious diseases that can be prevented through vaccination, including respiratory diseases and invasive bacterial diseases [1].

The implementation of vaccination in children undergoing cardiac surgery is suboptimal. Studies show that children with congenital heart defects who have undergone cardiac surgery have significant delays in vaccination, which persist into school age [5]. Similarly, the implementation of recommended vaccines against influenza and *S. pneumoniae* in adult patients belonging to risk groups is suboptimal and often falls significantly below targeted vaccination coverage thresholds [6,7,8,9,10,11,12].

In their analysis of a large, nationally representative sample of patients with heart failure in France, Roubille et al. identified age as a significant factor in pneumococcal (PCV13) vaccination: patients were less likely to be vaccinated as they grew older, with the odds of vaccination reduced by 52% in those older than 85 years, relative to younger patients [13].

### 3.2. Comorbidities

Patients undergoing cardiac surgery often present with multiple comorbidities, further increasing their risk of infectious complications. This underscores the importance of ensuring optimal protection against vaccine-preventable diseases through appropriate immunization strategies.

In children with congenital heart defects, additional factors that increase the risk of infectious diseases include low birth weight, prematurity, bronchopulmonary dysplasia and neurodevelopmental disorders [14]. Among adult patients with acquired cardiovascular diseases, common comorbidities are hypertension, diabetes, obesity, a history of smoking, and lung diseases [15,16].

### 3.3. Hospitalization Risks

Hospitalization is a known risk factor for infections, with specific implications for cardiac patients [17]. A preoperative hospital stay exceeding 3 days is associated with increased risk of nosocomial infections [18]. The overall length of hospitalization is also a risk factor for hospital-acquired infection in both the youngest and older age groups [19,20]. While the most common hospital-acquired pathogens are Gram-positive cocci, such as Staphylococcus aureus (*S. aureus*), a significant portion of infections involve other potentially vaccine-preventable pathogens, such as Streptococcus pneumoniae (*S. pneumoniae*). Hospital-acquired infections may also include viral infections that become complicated with bacterial superinfection, including antibiotic-resistant strains. This is especially concerning during the COVID-19, RSV, or influenza seasons [21].

The duration of hospitalization associated with cardiac surgery depends on the patient’s initial condition, the type of procedure and the presence of early complications [20]. In recent years, cardiac surgical procedures have become more complex [22,23]. Simultaneously, modern surgical techniques and protocols aimed at shortening recovery have developed. Efforts are being made to shorten the hospitalization period to as little as 3 days after surgery for patients with acquired cardiovascular diseases [24].

Once the patient’s clinical condition is stable, hospitalization should be considered as an opportunity to assess vaccination status and administer indicated vaccines or, at a minimum, provide evidence-based vaccination recommendations [25].

### 3.4. Additional Factors

#### 3.4.1. Blood Products Transfusion

Patients undergoing cardiac surgery may require transfusion of blood products. Both blood product transfusion [19,26] and postoperative hemorrhage requiring transfusion [27] have been identified as risk factors for infection in cardiac surgery patients of all ages.

Transfusions of blood products require careful consideration when planning an optimal vaccination strategy, as they may affect the efficacy of certain vaccines.

The antibodies present in blood products (e.g., whole blood, packed red blood cells and plasma) and in immune globulin preparations can inhibit the immune response to certain vaccines:

- Measles and rubella vaccines—inhibition for at least three months, the exact interval depending on the type and dose of the blood product. Detailed information is provided in the Vaccine Recommendations and Guidelines of the ACIP [28] (see “Timing and Spacing of Immunobiologics” within the General Best Practices for Immunization) and in Table 1 below.
- Mumps and varicella vaccines—the effect is uncertain, but commercial immune globulins do contain antibodies to these viruses, and the same rule therefore applies.
- Following the administration of antibody-containing products, measles, mumps and rubella (MMR) and varicella-zoster virus (VZV) vaccines should be delayed until the antibodies derived from those products have degraded, so as to avoid interference—except in high-risk situations, such as known exposure to a particular infectious disease. If antibody-containing products are required within 14 days after MMR or varicella vaccination, the vaccine dose should be repeated unless protective immunity is confirmed; more generally, whenever a live-virus vaccine is given too soon, a repeat dose is needed after the recommended interval.
- Yellow fever vaccine—interference is unlikely due to low antibody levels in blood products available in countries that are non-endemic for this disease, so in these regions there is probably no need to delay yellow fever vaccine.
- Other live and inactivated vaccines—there is no need to delay these vaccines after antibodies-containing blood products [28].

#### 3.4.2. Cardiopulmonary Bypass Procedure and Other Device-Based Support for Sustaining Organ Function

Cardiopulmonary bypass (CPB) during extracorporeal circulation may impact immunoglobulin G (IgG) antibody levels against infectious diseases. Moreover, CBP induces a systemic inflammatory response not only resulting from blood contact with artificial circuit surfaces but also due to ischemia–reperfusion injury, hypothermia and surgical trauma. This response involves complement activation, neutrophil and monocyte activation with cytokine release, transient lymphocyte dysfunction and subsequent postoperative immunosuppression. These mechanisms may contribute independently of vaccine-induced immunity [29].

The process of blood passing through CPB circuit, along with interventions like hypothermia and heparinization, can alter the body’s inflammatory response and potentially lower gamma globulin levels, similar to what is observed in hemodialysis [30]. However, the effect of CPB on post-vaccination antibody responses remains unclear. Previous studies have shown varied results. By measuring antibody titres before CPB and again the following morning, Vergales et al. found that CPB in children aged 2–14 months did not alter post-vaccination antibody levels for diphtheria, tetanus, polio, and Haemophilus influenzae type B (HiB), but was linked to lower titers for pertussis and hepatitis B [31]. This study, however, has several important limitations that are specific to the paediatric setting. The number of vaccine doses a child has received is determined by their age, which means that some patients had received fewer doses of the individual vaccines than others and that the interval between vaccination and surgery differed between patients. Furthermore, the developing immune system of a child may respond to vaccination differently from that of an adult, who may have acquired natural immunity over the course of life through prior infection. It should also be noted that antibody concentrations were measured immediately before and after surgery. Repeat assessment at a later time point—for example, three months postoperatively—would allow a more complete picture to be drawn, taking into account the transient decline in immune function attributable to the procedure itself. Notwithstanding these limitations, the authors conclude that, although antibody titres against pertussis and hepatitis B declined, this should not prompt any change in vaccination strategy in children undergoing cardiac surgery.

Conversely, Hayashi et al. observed a significant reduction in antibody levels against SARS-CoV-2 after CPB in adults [32]. These findings suggest that CPB may affect humoral immunity, although the magnitude of this effect appears to vary between diseases. There is no data about the CPB impact on immunological memory cells.

Multiple studies have shown that longer CPB duration is associated with an increased risk of infectious complications, including pneumonia [33] or *Enterobacteriaceae* invasive disease [34,35]. Among other evidence, analyses of COVID-19 vaccination, which has been extensively investigated over the last years, indicate that a decline in circulating antibody titres does not necessarily translate into a loss of clinical protection. Importantly, long-term immunity also depends on memory B cells, T-cell responses, and the functional quality of antibodies, none of which have been evaluated in the currently available studies [36].

The use of Extra Corporeal Membrane Oxygenation (ECMO); Intra-Aortic Balloon Pump (IABP); Continuous Renal Replacement Therapy (CRRT) and mechanical ventilation (MV) were indicated as risk factors for pneumonia following cardiac surgery [37]. Therefore, similarly to patients undergoing CPB, individuals at risk of requiring device-based support may benefit from optimization of their vaccination status before surgery.

Current data suggest that the CPB procedure may affect the persistence of vaccine-induced humoral immunity, but the studies are not sufficient to formulate recommendations regarding the need for potential revaccination or booster doses post-CPB.

In summary, when planning an optimal prophylaxis strategy with vaccination, all patients scheduled for cardiac surgery with CPB should be provided with maximum protection against invasive infections and respiratory tract infections.

**Figure 1:**
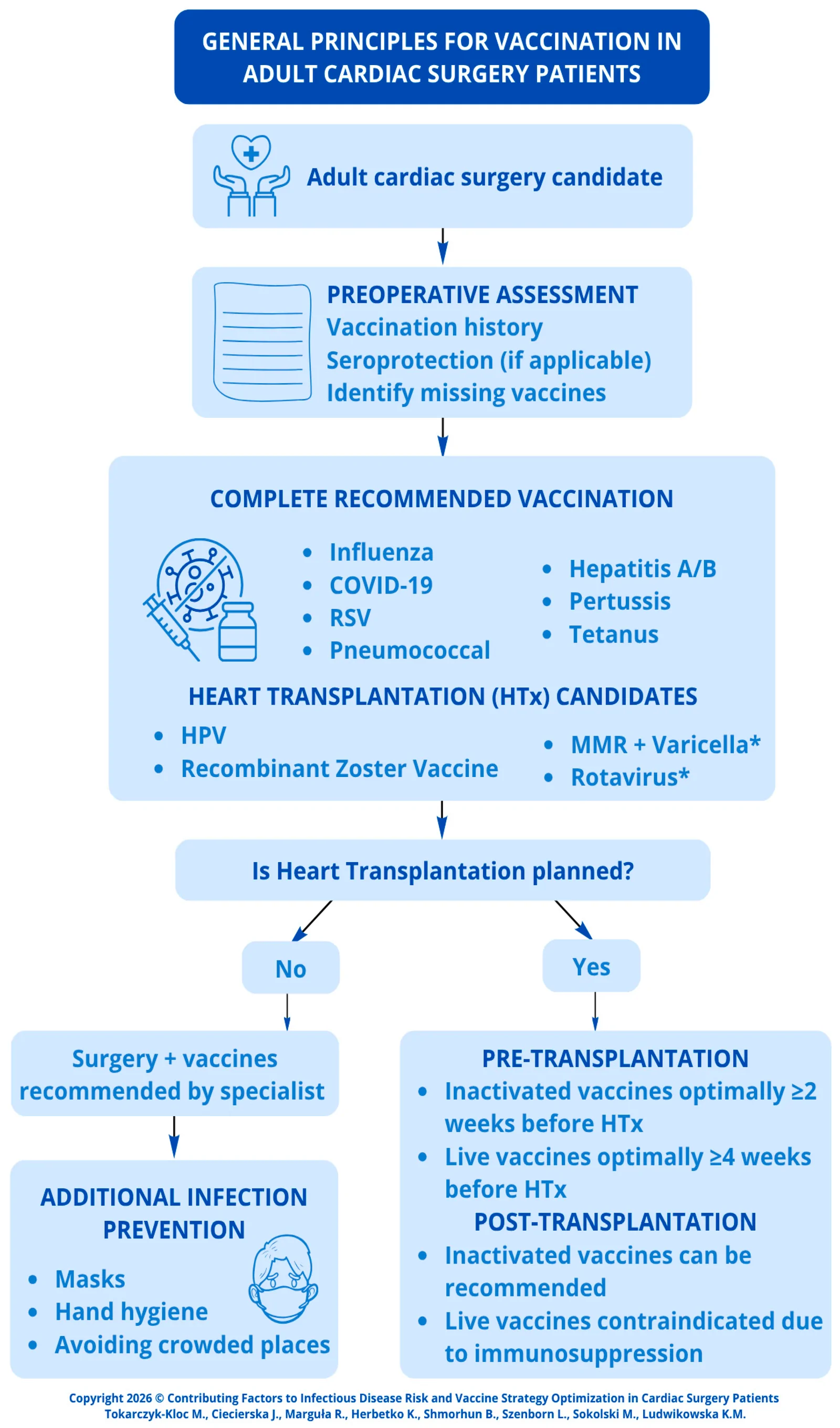
General Principles for Vaccination in Cardiac Surgery Patients. * Depending on the age and serological status of the patient.

General principles for vaccination in cardiac surgery patients are presented in Figure 1.

#### 3.4.3. Heart Transplantation (HTx)

As a general principle, candidates for HTx require broad protection against infectious diseases through vaccinations. Recommendations state that vaccination history and serological immunity status (when applicable) should be evaluated before heart transplant listing. Current ISHLT recommendations support completion of indicated vaccinations before transplantation as early as possible, as advanced organ dysfunction and medically induced immunosuppression can reduce vaccine efficacy. Patients can receive prior to transplantation both live-attenuated vaccines (when indicated and if they are seronegative against vaccine-preventable diseases, such as measles, mumps, rubella, varicella-zoster virus) and inactivated vaccines, including those against influenza, pneumococcal disease, RSV, tetanus, pertussis, hepatitis A and B, and HPV, following established guidelines. Ideally, these vaccines should be administered and completed at least two weeks before the HTx procedure for inactivated vaccines and at least four weeks before HTx for live vaccines [28,38]. If that is not feasible, inactivated vaccines can be used under immunosuppression, although their effectiveness may be lower than in immunocompetent individuals. Available evidence indicates reduced immunogenicity after transplantation; however, even partial protection is considered preferable to the absence of protection. Therefore, additional non-pharmacological protective measures are often recommended, such as avoiding crowded environments, especially during the infection season, wearing protective masks when avoidance is not possible, and practicing good hand hygiene. Live vaccines are contraindicated in immunosuppressed patients [28]. Further details on post-heart transplantation management are beyond the scope of this review and can be found in “The International Society for Heart and Lung Transplantation (ISHLT) guidelines for the care of heart transplant recipients” [38,39] (see Table 2 below).

It is important to note that neither planned nor completed surgical procedures, including cardiac surgery, constitute contraindications for administering any vaccination at any age [40].

Vaccine safety in patients requiring heart surgery is comparable to that of the general population, and the benefits of vaccination outweigh the risks of adverse effects. These effects are usually mild and last no more than 3 days and such a message is promoted by major medical associations and organizations [41]. However, to avoid uncertainties in interpreting adverse reactions post-vaccination, it is advisable to avoid inactivated vaccines 3–5 days before the planned procedure and live vaccines 7–21 days before the planned procedure. These time frames are based on the period after vaccination during which fever may be expected, depending on the type of vaccine. This should be considered as a precaution, not a contraindication. The decision to vaccinate should be made in consultation with the multidisciplinary care team, including the cardiac surgeon, anesthesiologist, cardiologist, and, when appropriate, a specialist in infectious diseases, clinical immunology, or vaccinology.

## 4. Discussion

Chronically ill patients, including those requiring cardiac surgery, are particularly vulnerable to infectious diseases and should therefore be prioritized for preventive interventions.

### 4.1. Vaccine Coverage

Influenza vaccination coverage for at-risk groups remains significantly below the WHO/EU target of 75% [6]. The WHO European region reported that influenza vaccination coverage rates for individuals with chronic conditions are mostly below 40% across 14 European countries [42]. Pneumococcal vaccination coverage also varies widely, with reported rates ranging from as low as 7% in Italy to up to 50% among immunocompromised patients in the United States, and 60% in high-risk groups in Catalonia, Spain [9,10,11,12].

Higher coverage rates were observed in patients aged over 65 years (60% for influenza and 36% for *S. pneumoniae*), while younger age groups with non-age-related risk factors had lower coverage (28% for influenza and 16% for *S. pneumoniae*) [10].

Summarizing the above issues, it becomes clear that patients qualified for cardiac surgery are at high risk of infectious diseases. In recent years, vaccinology associations and advisory committees have focused their vaccination recommendations on more narrowly defined high-risk groups. However, cardiac surgical patients remain an uncovered population in this regard. Several recommendations specifically address patients with cardiovascular diseases, while Australian guidelines specify the issue of planned surgery in the context of vaccine interference and identify cardiac surgery with extracorporeal circulation as a factor that may potentially attenuate the post-vaccination response.

The key to understanding the rationale behind protective measures is educating the patient about the risks associated with contracting a particular disease and how to protect against infection—using non-specific methods, such as hand hygiene, protective equipment and avoiding contact with sick individuals, as well as specific methods, including vaccinations.

### 4.2. Clinical Implementation of Vaccination Process

One potential factor contributing to these low vaccination coverage rates is that high-risk patients, who are often monitored by specialists, may not consult general practitioners—the primary administrators of vaccines in most countries—as frequently as the general population. Specialists typically do not address vaccination with patients, as this responsibility is generally assigned to general practitioners; a vaccination recommendation from a specialist could significantly improve vaccination rates [42,43]. The recent ACC Expert Consensus Statement on Adult Immunizations as Part of Cardiovascular Care explicitly identifies cardiology clinics as an important opportunity for vaccination, since patients may find it easier to discuss how vaccination fits into their treatment plan with their cardiologist than with a primary care physician [44]. Assessment of vaccination status should become a routine component of the preoperative evaluation of cardiovascular patients undergoing cardiac surgery, similarly to perioperative risk assessment and evaluation of clinical status. When necessary, patients should be referred for consultation with an infectious diseases specialist to determine the optimal vaccination strategy and to facilitate vaccination at the earliest appropriate time after surgery.

In Boey et al.’s study investigating vaccination coverage and determinants of vaccination among at-risk patients in Belgium (including patients with heart failure and recipients of solid organ transplants among others), 71% of the studied group reported having received information about influenza vaccination. Among these patients, 60% obtained their information from their general practitioner, while 30% received it from a specialist. For pneumococcal vaccination, 29% of patients reported being informed about the vaccine. Of these, 48% were informed by their general practitioner, and 47% by a specialist. Other sources of information for both influenza and pneumococcal vaccinations were occupational health professionals, family, and friends. Notably, fewer than 10% of patients with chronic conditions received all recommended vaccinations [45]. Researchers investigating reasons for not receiving specific vaccines in solid organ transplant patients, including 127 heart transplant recipients and 200 patients with heart failure, found that they were varied and vaccine dependent. For diphtheria–tetanus vaccination, the most common reasons were a lack of awareness about the recommendation (38%) and forgetting to get the vaccine (29%). Regarding influenza vaccination, 41% of individuals reported that they intended to receive it, while others cited concerns about the vaccine’s safety (13%), necessity (6%), and effectiveness (6%), or were opposed to influenza vaccination (9%). For pneumococcal vaccination, a significant 89% were unaware of the recommendation [46].

The study cited earlier found that, despite frequent contact with nurses, general practitioners and specialists, only 10% of patients had received pneumococcal vaccination in 2020, even though the vaccine was provided free of charge [13]. This points to the need for healthcare professionals to remind patients continually of the importance of vaccination and to monitor immunisation status in every patient.

Sense of security and understanding constitute the most important foundations of any vaccination programme’s efficiency. These foundations can be strengthened by the active participation of healthcare workers and their willingness to provide patients with the time that is indispensable for explaining how vaccination works and demonstrating its effectiveness. Numerous studies have shown that efforts by healthcare workers to increase vaccine confidence play an essential role in improving the uptake of a range of vaccines. Physicians, nurses and physiotherapists are among the most trusted and important sources of health education for patients, and their collaborative effort therefore has considerable potential to dispel the misconceptions that underlie vaccine hesitancy [47,48,49]. For this reason, it is of great importance to raise the awareness of all healthcare workers as to the influence they have on patients, since they remain the most trusted advisors and the most prominent influences on any decision relating to vaccination [50]. They should therefore adopt an open attitude and be properly trained to address questions about vaccines and to discuss their risks and benefits.

**Figure 2:**
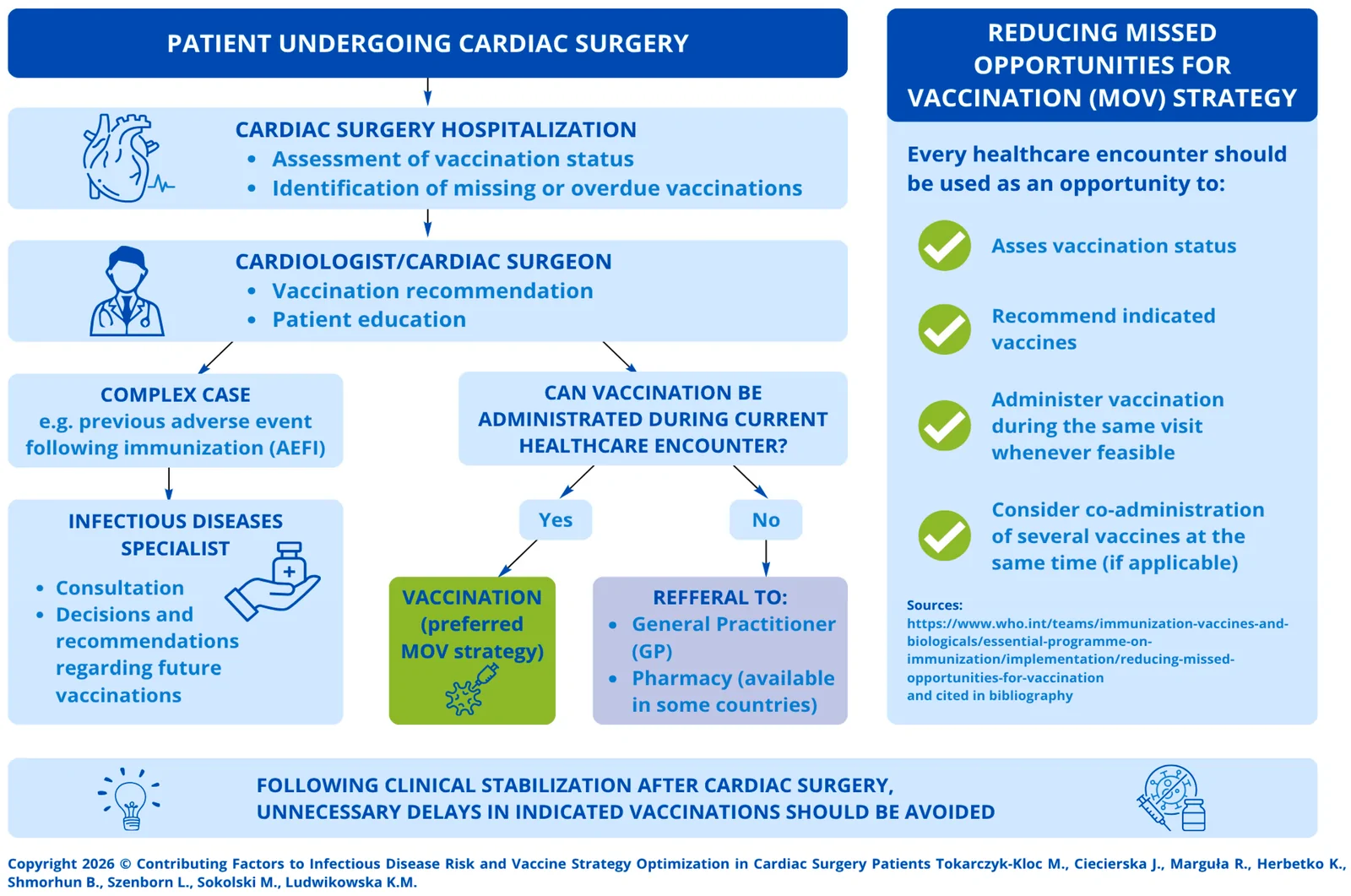
Decision-making strategies for reducing Missed Opportunities for Vaccination.

In light of the data presented above, the authors of this manuscript consider that vaccination should be recommended to patients by specialists—namely cardiologists and cardiac surgeons—and ideally administered during hospitalisation, once the patient’s condition has stabilised, thereby avoiding a missed opportunity for vaccination. Where this preferred pathway is not feasible, vaccination should instead be carried out by the general practitioner, at an infectious diseases clinic or, depending on availability and local policy, in community pharmacies and long-term care facilities. In cases of uncertainty, a complex medical history or previous adverse effects, the specialist may refer the patient for consultation with an infectious disease specialist, who will then decide on vaccination. Such collaborative care is essential if patients are to be afforded optimal protection against vaccine-preventable diseases. The approach proposed by the authors is presented in Figure 2.

At the same time, the cocoon strategy should not be overlooked. This approach involves vaccinating immunocompetent individuals in the immediate environment of a patient who, for any of a number of reasons, cannot be vaccinated themselves. In the heart transplant recipients discussed in the present review, immunosuppressive therapy precludes the administration of live vaccines; offering these vaccines to close contacts instead may therefore contribute to better protection of the patient [51]. This subject is addressed in detail by the Infectious Diseases Society of America (IDSA) in its Clinical Practice Guideline for Vaccination of the Immunocompromised Host. The guideline recommends that healthy, immunocompetent individuals sharing a household with an immunocompromised patient receive inactivated vaccines in line with the annually updated CDC–ACIP schedules—for example, against influenza and COVID-19—as well as certain live vaccines according to the CDC annual schedule, namely combined measles, mumps and rubella (MMR) vaccine, rotavirus vaccine in infants aged 2–7 months, varicella vaccine and zoster vaccine. Such household contacts may also safely receive the yellow fever and oral typhoid vaccines for travel. Oral polio vaccine (OPV), by contrast, should not be administered to anyone living in a household with an immunocompromised patient [52]. It should be noted, however, that although this guideline remains in force, it was published in 2013, and the evidence on which its recommendations rest may since have been superseded in places.

Like any other medicinal product, vaccines are capable of causing adverse effects. Concern about these effects is a common source of patient anxiety and contributes to vaccine hesitancy and, ultimately, to vaccine refusal. Adequate knowledge and preparation on the part of healthcare staff are therefore essential, as they enable clinicians to address these concerns directly in conversation with patients. A readiness to report any adverse effects in accordance with local protocols is equally important: it underpins the reliability of vaccine safety data, which provides the evidence base for those same conversations. Organisations such as immunize.org can serve as a reliable source of information for healthcare providers, enabling them to build their own knowledge that can then be shared with patients [53].

The fact that the available recommendations do not clearly define the respective responsibilities of the cardiologist, the cardiac surgeon and the general practitioner further underscores the need for the harmonised, consensus-based approach that this review seeks to support.

### 4.3. Minimizing Missed Opportunities for Vaccination (MOV)

The World Health Organization defines a missed opportunity for vaccination (MOV) as “any contact with health services by an individual (child or person of any age) who is eligible for vaccination (e.g., unvaccinated or partially vaccinated and free of contraindications to vaccination), which does not result in the person receiving one or more of the vaccine doses for which he or she is eligible”. Observation of this phenomenon led the WHO to introduce a strategy aimed at reducing such missed opportunities [54].

Hospitalised patients are frequently among those in need of immunisation—not least because the admission itself may result from deterioration of the underlying condition brought on by a vaccine-preventable infection. Yet, advice issued by specialists to complete vaccination through the family doctor is acted upon by patients too rarely. To close this gap, the Community Preventive Services Task Force (CPSTF) recommended the use of standing orders in 2015 under which healthcare staff—nurses, pharmacists and other providers—are authorised to assess a patient’s vaccination status at every contact with health services and to administer missing doses in accordance with local protocols [55]. Implementing such a strategy is organisationally demanding: it calls for the training of large numbers of staff and for secure funding of the vaccines themselves, both of which remain challenging for local management and national health system policies.

The scale of the problem is illustrated by a retrospective cohort study from Italy, which found that missed opportunities reduce seasonal influenza vaccine coverage among high-risk older adults who access healthcare services frequently but remain unvaccinated. Beyond documenting the under-provision of influenza vaccination during hospitalisation, the authors draw attention to the question of co-administration: although uptake was suboptimal overall, among the doses that were given it was most frequent in the influenza–pneumococcal and influenza–RSV cohorts—a pattern that may translate into effective protection against respiratory tract infections [56].

The clinical benefit of such co-administration is supported by a prospective randomised trial in high-risk heart failure patients attending a hospital-based outpatient clinic. Simultaneous influenza and RSV vaccination, compared with standard of care, significantly reduced the primary outcome, an effect driven by a reduction in infections [43].

Cardiac patients thus stand to derive particular benefit from vaccination, and all healthcare providers involved in their care should make every effort to reduce missed opportunities for vaccination in this group (as presented in Figure 2).

### 4.4. Vaccine Hesitancy

According to the definition adopted by the WHO SAGE Working Group on Vaccine Hesitancy in 2015, vaccine hesitancy is a “delay in acceptance or refusal of vaccines despite availability of vaccination services” [57] and it has been listed by the WHO among the ten leading threats to global health [58,59].

Despite the availability of vaccination services, of reliable information on their effectiveness and of clear medical indications for their use, vaccine hesitancy remains a growing problem, fuelled by disinformation that is frequently politically affiliated and driven by the pursuit of social polarisation and financial gain [60,61].

A report on medical disinformation published in June 2026 by Wroclaw Medical University in collaboration with Mediaboard Polska found that anti-vaccination narratives accounted for the largest single share of medical disinformation in the Polish media, at 54% of the content analysed between January 2025 and May 2026 [62].

At the same time, recommendation from a healthcare professional is consistently identified as one of the strongest predictors of vaccine uptake [44,63,64]. This makes it all the more important that the position taken by the medical community should rest on rigorous scientific analysis and on the principles of evidence-based medicine. Vaccination is among the most effective and most extensively studied of all public health interventions, and the recent statements issued by scientific societies reinforce this message unequivocally.

### 4.5. Summary

It is reasonable to hypothesise that vaccine coverage in patients with chronic heart disease requiring cardiac surgery would be higher if unified recommendations were in place, together with greater awareness among healthcare professionals of the vaccines that should be administered to minimise the risk of infection. Such guidance has been clearly formulated for heart transplant recipients. For patients undergoing procedures involving cardiopulmonary bypass, however, our review found no official recommendations; the bypass is mentioned only in the Australian guidelines, as a factor that may potentially attenuate the post-vaccination response. The discrepancies between the positions of the major vaccinology societies are summarised in Table 2.

**Table 2:** Summary of published vaccination guidelines developed by various vaccinology societies.

Name	Country	Summary of Recommendations Regarding Patients with Heart Diseases and Requiring Cardiac Surgery	Comments
		By scientific organization	
The American College of Cardiology (ACC), 2025 [43]	USA	Influenza—annual for persons aged ≥6 months; nasal vaccine not recommended for patients aged > 50 years. Specific vaccines are recommended for those aged ≥65 years. Pneumococcal—1-time vaccination with PCV20 or PCV21 for adults aged ≥19 years, other vaccines require 2 vaccinations COVID-19—Initial and 2024–2025 vaccine for persons aged ≥6 months; recommendations for annual vaccination may change RSV—1-time vaccination for adults aged ≥60 years Zoster—2 doses of recombinant zoster vaccine 2–6 months apart for those adults aged ≥50 years [44]	The recent ACC consensus statement provides clinicians with clear recommendations on vaccination in patients with heart disease, based on the relevant, evidence-based recommendations of the American Heart Association (AHA) and the Centers for Disease Control and Prevention (CDC). It does not, however, contain any separate provisions for patients being assessed for or recovering from cardiac surgery, including heart transplantation, or any addressing cardiopulmonary bypass.
The International Society for Heart and Lung Transplantation (ISHLT), 2023	USA	Every attempt should be made to complete live virus vaccines, including MMR, varicella, live-attenuated zoster, and rotavirus, before transplantation in non-immune patients according to established guidelines. Live virus vaccination should ideally be completed four weeks before transplantation. Before transplantation, candidates should receive inactivated vaccines, including but not limited to influenza, pneumococcal, tetanus, pertussis, hepatitis A and B, and human papillomavirus vaccines, in accordance with established guidelines. Inactivated vaccines should ideally be completed two weeks before transplantation. The recombinant subunit zoster vaccine is preferred over the live-attenuated vaccine for transplant candidates and should be given in accordance with local vaccination guidelines. COVID-19: Pre-transplant vaccination of all solid organ transplant (SOT) candidates as a priority whenever feasible. Vaccination in SOT recipients and priority for vaccination of their household members and caregivers to reduce exposure risk for these vulnerable patients. Live viral vaccines even if attenuated should be avoided. Use of vaccines with mRNA technology is safe in immunocompromised patients but efficacy may be reduced. Reduced efficacy of new vaccines in the immunocompromised transplant recipient should be considered during a pandemic crisis and proper education given on need to minimize exposure risks despite vaccination. Based on current evidence, providing a third dose of mRNA vaccine for SOT recipients that have previously completed a 2-dose mRNA vaccine series is recommended. The use of repeated booster vaccines should be supported as further evidence is available. Ongoing booster vaccination should be based on the individual patient’s unique situation and may depend on local availability of vaccines and local regulations. HPV: Individuals 9–45 years should receive all 3 doses before HT if possible. There is no contraindication to administering the vaccine to women after HT, although studies have shown decreased immunogenicity with quadrivalent HPV vaccination in adult solid organ transplant recipients Travel medicine: Live-viral vaccinations should NOT be given to heart transplant recipients for travel (Live-attenuated influenza vaccine (LAIV), oral typhoid, oral polio, varicella or live zoster vaccine, MMR, BCG, or Yellow Fever; live-attenuated Japanese Encephalitis (JE) Virus vaccine not recommended, but inactivated JE vaccine is recommended) [38].	The International Society for Heart and Lung Transplantation (ISHLT) provides highly detailed recommendations on vaccination both for candidates for heart transplantation and following transplantation, including guidance on travel vaccines. The COVID-19 vaccination recommendations in these guidelines, drawn up in 2022, were based on the evidence available at that time. Given how rapidly knowledge in this area has developed since then, this section would in all likelihood benefit considerably from updating. Here as well, however, no consideration is given to the influence of cardiopulmonary bypass on any modification of the vaccination schedule.
Centers for Disease Control and Prevention (CDC) and Advisory Committee on Immunization Practices (ACIP)	USA	ACIP General Vaccine Recommendations provide broad vaccine protection advice including COVID-19, influenza, RSV, pneumococci, Tdap/Td, MMR, chickenpox, shingles, HPV, HAV, HBV, meningococci, HiB and Mpox [65]. General seasonal influenza vaccination recommendation specifying cardiovascular diseases as an indication for vaccination among other risk factors for severe influenza [66] Patient information with recommendations of vaccines for adult patients with heart diseases, highlighting influenza and pneumococcal vaccines necessity [65].	These guidelines present vaccination recommendations for adults in a systematic manner; they do not, however, contain any specific provisions for patients being assessed for cardiac surgery.
European Society of Cardiology (ESC), 2025	Europe	Influenza—annually, before winter; possible in acute conditions (e.g., acute HF); possibility of simultaneous vaccination Pneumococcal—every 5–10 years; possible in acute conditions; possibility of simultaneous vaccination COVID-19—possible in acute conditions (e.g., acute HF); possibility of simultaneous vaccination RSV—every 2 years; possibility of simultaneous vaccination Zoster—according to the vaccination schedule Diphtheria, tetanus–polio—according to the vaccination schedule Advised vaccinations before transplant: Measles, mumps and rubella (MMR), Varicella, Herpes zoster, Rotavirus, COVID-19, Influenza, Pneumococcus, Tetanus, pertussis, Hepatitis A and B, HPV [67]	The ESC addresses the group of patients undergoing heart transplantation in detail in its recommendations. There is a lack of clear guidance on the intervals at which the COVID-19 and RSV vaccines should be administered.
		By country	
Canadian Immunization Guide (CIG)	Canada	The recommendations for patients with cardiovascular disease include influenza vaccination annually, pneumococcal vaccination (with PCV-15 or PCV-20) from the age of 50, and RSV vaccination in adults aged 75 and over. Different vaccines are described in context of safety of their use in cardiological patients. Vaccination of solid organ transplant (SOT) recipients—covering both the pre- and postoperative periods—in a single, detailed section [68,69]	These guidelines are set out in a particularly clear and accessible manner. They also address, in the context of patients with heart disease, several vaccines not covered by the other guidelines, including those against cholera and travellers’ diarrhoea, rabies, typhoid, tuberculosis (BCG) and smallpox, among others. Patients undergoing cardiac surgery are not addressed separately.
National Health Service (NHS)	UK	Influenza—annually Pneumococcal—PPV-23 [70] Additionally, for people on immunosuppresive therapy also the vaccine against Zoster is recommended [71]	UK Health Security Agency in their Complete routine immunisation schedule lists chronic heart diseases next to chronic pulmonary diseases as additional vaccines for patients with medical conditions; patients undergoing cardiac surgery are not addressed separately
Australian Technical Advisory Group on Immunisation (ATAGI)	Australia	The guidelines classify chronic heart disease as a mild immunocompromising condition, defined by an increased risk of viral and bacterial infection arising from organ dysfunction rather than from immunosuppression in the strict sense. The degree of immunocompromise is greater, however, in patients who have recently undergone extracorporeal membrane oxygenation. Patients with chronic heart diseases and undergoing cardiac surgery are not addressed separately Patients before surgery and anesthesia are addressed- optimal timeframe for avoidance of overlapping adverse reactions and postsurgical period whenever possible is recommended; blood products administration impact on vaccines response is described as well [71,72]	The Australian guidelines address the issue of planned surgery in the context of vaccine interference and identify cardiac surgery with extracorporeal circulation as a factor that may potentially attenuate the post-vaccination response.
Taiwan Society of Cardiology and the Infectious Diseases Society of Taiwan	Taiwan	Influenza: recommended annually for patients with chronic coronary syndrome, HF, for early post-MI patients COVID-19 and pneumococcal: recommended for patients with cardiovascular disease (CVD) RSV—recommended for adults aged ≥75 years and those aged 50–74 years with established CVD, chronic lung disease, or living in nursing homes Zoster—adjuvanted recombinant zoster vaccine is recommended for adults aged ≥50 years and those aged 18–49 years with CVD Diphtheria toxoid-, tetanus toxoid-, and acellular pertussis-containing vaccine—general recommendation for adults aged ≥65 years in Taiwan [73]	These detailed guidelines cover only some of the vaccinations relevant to cardiology patients and are consistent with the American recommendations; they do not address patients being assessed for cardiac surgery.
Polish Vaccinology Association (Polskie Towarzystwo Wakcynologiczne)	Poland	The Polish Vaccinology Association has created vaccine schedules for particular risk groups, a schedule for patients with underlying heart disease includes recommendation of influenza, pneumococci, Tdap, COVID-19, RSV and Zoster; patients undergoing cardiac surgery are not addressed separately [74].	Patients undergoing cardiac surgery are not addressed separately

In preparing this manuscript, the authors reviewed a broad range of guidelines and selected those judged most relevant to the questions considered here. We acknowledge that this selection, based on the authors’ knowledge and clinical experience rather than on a predefined systematic search strategy, is a limitation of the present work.

To sum up, vaccine coverage among patients with chronic cardiovascular diseases, including those undergoing cardiac surgery and heart transplantation, is suboptimal and should be improved. The main obstacles to vaccination appear to be the lack of specific recommendations, insufficient knowledge about vaccination indications resulting from the absence of recommendations from healthcare providers managing the patient, and the lack of effective reminder systems for vaccination needs. Among the multitude of medical instructions received by cardiac surgery patients, vaccination information may be overlooked, resulting in missed opportunities for immunization or its suboptimal implementation.

## 5. Conclusions

In conclusion, patients undergoing cardiac surgery require a detailed approach regarding appropriate immunization, thus avoiding severe infectious diseases and their complications.

The interval between vaccination and heart surgery is unrestricted except for the patients who have planned immunosupressive treatment and live vaccines. As a precaution, overlapping of possible vaccine adverse reactions and surgical complications should be avoided (up to 3–4 days after inactivated vaccines and between 7 and 14 days after live vaccines). For HTx inactivated vaccines should be completed two weeks before surgery, and with live vaccines four weeks before surgery and immunosupressive treatment.

Vaccines particularly recommended for cardiac surgery patients include those against influenza, COVID-19, pneumococcal disease, tetanus, pertussis, RSV, hepatitis A and B, and for immunosuppresed patients after HTx additionally against HPV and VZV. One should also not forget to recommend non-specific means of protection against infections to patients—frequent hand washing, avoiding large crowds, and wearing protective masks. The presented methods can contribute to avoiding infections with severe courses, and thus to improving the quality of life of patients after major cardiac surgeries.

We propose that assessment of vaccination status should become a routine component of preoperative cardiac surgical evaluation and in case of lacking appropriate vaccinations, these should be supplemented as quickly as possible. In our opinion, cardiac surgery represents an opportunity to optimize immunization, rather than contraindication for vaccination.

There is a clear knowledge gap concerning explicit, evidence-based recommendations for patients undergoing cardiac surgery. Further research and concerted effort are needed to ensure that no opportunity to protect these vulnerable patients is missed.

## Figures and Tables

**Table 1:** Suggested intervals between administration of immunoglobulin, blood, other blood products and measles or chickenpox vaccination.

Products Containing Antibodies	Dose * (Including IgG)	Interval (Months)
Monoclonal antibodies against RSV (Synagis or Nirsevimab)	15 mg/kg i.m. for palivizumab and 50 or 100 mg depending on body weight for nirsevimab	not applicable
Tetanus antitoxin	250 IU (10 mg IgG/kg) i.m.	3
Hepatitis A prophylaxis		
- after contact	0.02 mL/kg (3.3 mg IgG/kg) i.m.	3
- before travelling abroad	0.06 mL/kg (10 mg IgG/kg) i.m.	3
Hepatitis B prophylaxis	0.06 mL/kg (10 mg IgG/kg) i.m.	3
Rabies prophylaxis	20 IU/kg (22 mg IgG/kg) i.m.	4
Measles prophylaxis		
- standard doses	0.25 mL/kg (40 mg IgG/kg) i.m.	5
- in immunocompromised patients	0.50 mL/kg (80 mg IgG/kg) i.m.	6
Blood transfusions		
- flushed red cells	10 mL/kg IgG/kg i.v.	not applicable
- red cell mass	10 mL/kg (60 mg IgG/kg) i.v.	6
- whole blood	10 mL/kg (80–100 mg IgG/kg) i.v.	6
- platelet mass	10 mL/kg (160 mg IgG/kg) i.v.	7
Products against CMV infection	150 mg/kg maximum i.v.	6
IVIG		
- substitution therapy and treatment of immunodeficiencies	300–400 mg/kg i.v.	8
- post-exposure prophylaxis of chickenpox	400 mg/kg i.v.	8
- primary immune thrombocytopenia	400–1000 mg/kg i.v.	10
- Kawasaki disease	2 g/kg i.v.	11

* Dosages indicated per kg refer to the patient’s body mass. Source: General recommendations on immunisation. Recommendations of the Advisory Committee on Immunization Practices (ACIP). MMWR Recomm. Rep., 2011; 60: 1–64 [29]. CMV—cytomegalovirus, IVIG—intravenous immunoglobulin preparation.

## Data Availability

No new data were created or analyzed in this study. Data sharing is not applicable to this article.

## Abbreviations

ACC	American College of Cardiology
ACIP	Advisory Committee on Immunization Practices
ACVC	Association for Acute Cardiovascular Care
AHA	American Heart Association
CDC	Centers for Disease Control and Prevention
CPB	Cardiopulmonary Bypass
COVID-19	coronavirus disease 2019
EAPC	European Association of Preventive Cardiology
ESC	European Society of Cardiology
HF	heart failure
HFA	Heart Failure Association (of the ESC)
HTx	Heart Transplant
HPV	human papillomavirus
IDSA	Infectious Diseases Society of America
ISHLT	International Society for Heart and Lung Transplantation
MACE	major adverse cardiovascular events
MMR	measles, mumps and rubella (vaccine)
MOV	missed opportunity for vaccination
PCV13	13-valent pneumococcal conjugate vaccine
RSV	respiratory syncytial virus
RTI	respiratory tract infection
SAGE	Strategic Advisory Group of Experts (on Immunization)
SOC	standard of care
SOT	solid organ transplant
VZV	varicella-zoster virus
WHO	World Health Organization
